# Nomenclature for factors of the HLA system, update December 2009

**DOI:** 10.1111/j.1399-0039.2010.01462.x

**Published:** 2010-07

**Authors:** Steven G E Marsh, for the WHO Nomenclature Committee for Factors of the HLA System

The following sequences have been submitted to the Nomenclature Committee since the November 2009 nomenclature update and, following agreed policy, have been assigned official allele designations. Full details of all sequences will be published in a forthcoming report.

Below are listed the newly assigned sequences (Table 1) and confirmations of previously reported sequences (Table 2). The accession number of each sequence is given and these can be used to retrieve the sequence files from the EMBL, GenBank, Vega or DDBJ data libraries. Although accession numbers have been assigned by the data-libraries and most sequences are already available, there is still the possibility that an author may not yet have allowed the sequence to be released; in such a case you will have to contact the submitting author directly. Additional information pertaining to new sequences is often included in the publications describing these alleles; a listing of recent publications that describe new HLA sequences is given in Table 3.

**Table 1:** New Sequences

Sequence	Cell identification	Accession number	Submitting author
A*0372	JMDP01K043	AB535153	Kenji Tadokoro, Tokyo, Japan
A*0373	BY00578	GU256011	Carolyn K Hurley, Washington DC, USA
A*0374	BY00575	GU256014	Carolyn K Hurley, Washington DC, USA
A*2511	BY00577	GU256012	Carolyn K Hurley, Washington DC, USA
A*300205	BY00580	GU256009	Carolyn K Hurley, Washington DC, USA
A*3033	JMDP01K045	AB535154	Kenji Tadokoro, Tokyo, Japan
A*3034	BY00564	GU138062	Carolyn K Hurley, Washington DC, USA
A*3221	BY00581	GU256008	Carolyn K Hurley, Washington DC, USA
A*3605	170705	FN557302	Elizabeth Keller, Munich, Germany
A*927102	BY00576	GU256013	Carolyn K Hurley, Washington DC, USA
B*1336	JMDP36K066	AB536746	Kenji Tadokoro, Tokyo, Japan
B*400209	JMDP36K061	AB536744	Kenji Tadokoro, Tokyo, Japan
B*5189	JMDP36K062	AB536745	Kenji Tadokoro, Tokyo, Japan
B*5828	BY00573	GU256006	Carolyn K Hurley, Washington DC, USA
B*9586	JMDP36K033	AB477102	Kenji Tadokoro, Tokyo, Japan
Cw*0629	OUAL	FN597419	Valerie Dubois, Lyon, France
Cw*1526	SZBM685	GU232859	Zhihui Deng, ShenZhen, China
DRB1*040104	HN-88028-8	FJ438928	Histogenetics, Ossining, USA
DRB1*040105	HN-20315-2	FJ438930	Histogenetics, Ossining, USA
DRB1*040106	HN-57737-8	FJ549416	Histogenetics, Ossining, USA
DRB1*040305	HN-09532-5	FJ438927	Histogenetics, Ossining, USA
DRB1*040403	HN-37506-5, HN-78268-8	FJ438924, FJ594733	Histogenetics, Ossining, USA
DRB1*040404	HN-00728-6	FJ438929	Histogenetics, Ossining, USA
DRB1*040509	HN-8629540	FJ766004	Histogenetics, Ossining, USA
DRB1*040704	HN-18682-5, HN-763322	FJ438923, FJ489881	Histogenetics, Ossining, USA
DRB1*0489	HN-6790922	FJ858914	Histogenetics, Ossining, USA
DRB1*115802	HN-31092-9	FJ766005	Histogenetics, Ossining, USA
DRB1*130304	HN-64842-5	FJ858909, FJ858910	Histogenetics, Ossining, USA
DRB1*132102	HN-50459-4, HN-64798-8, HN-65133-7, HN-55271-7, HN-50682-0, HN-57306-9	FJ766012, FJ858912, FJ875605, FJ875606, FJ875607, FJ858911	Histogenetics, Ossining, USA
DRB1*1395	HN-67038-8	FJ640587	Histogenetics, Ossining, USA
DRB1*1396	HN-66141-1	FJ640586	Histogenetics, Ossining, USA
DRB1*1397	HN-26392-8	FJ766011	Histogenetics, Ossining, USA
DRB1*150110	HN-30368-4	FJ358155	Histogenetics, Ossining, USA
DRB1*150111	HN-55222-3, HN-62717-0	FJ358157, FJ875603	Histogenetics, Ossining, USA
DRB1*150112	HN-17926-7	FJ358169	Histogenetics, Ossining, USA
DRB1*1542	HN-39373-2	FJ766007	Histogenetics, Ossining, USA
DRB1*1543	HN-44736290	FJ858913	Histogenetics, Ossining, USA

**Table 2:** Confirmatory Sequences

Sequence	Cell identification	Accession number	Submitting author
A*030104	JMDP36K031	AB536869	Kenji Tadokoro, Tokyo, Japan
A*0357	P-661594	FN430729	Rainer Blasczyk, Hannover, Germany
A*2405	JMDP01K069	AB536870	Kenji Tadokoro, Tokyo, Japan
A*2426	BY00562	GU138064	Carolyn K Hurley, Washington DC, USA
A*6806	BY00563	GU138063	Carolyn K Hurley, Washington DC, USA
A*6807	BY00567	GU144508	Carolyn K Hurley, Washington DC, USA
A*680802	BY00579	GU256010	Carolyn K Hurley, Washington DC, USA
A*9282	AKB-650304	FN430730	Rainer Blasczyk, Hannover, Germany
B*070208	HN-46788-1, HN-12328-3	GQ449646, GQ859541	Histogenetics, Ossining, USA
B*070210	HN-97825-6	GQ994063	Histogenetics, Ossining, USA
B*0777	HN-57433-7	GQ401200	Histogenetics, Ossining, USA
B*0780	HN-24661-7	GQ914797	Histogenetics, Ossining, USA
B*0787	HN-30108-0, HN-84250-6, HN-30107-2, HN-20795-6	GQ245737, GQ254338, GQ345063, GQ994071	Histogenetics, Ossining, USA
B*080107	HN-85124-4	GQ254351	Histogenetics, Ossining, USA
B*080108	HN-82787-0, HN-12663-3, HN-60847-6	GQ240393, GQ449633, GQ994057	Histogenetics, Ossining, USA
B*080108	P-664367	FN430731	Rainer Blasczyk, Hannover, Germany
B*081203	HN-98041-3	GQ240392	Histogenetics, Ossining, USA
B*0841	HN-98338-7, HN-97818-9	GQ199710, GQ254343	Histogenetics, Ossining, USA
B*1418	HN-03501-0	GQ245747	Histogenetics, Ossining, USA
B*150303	HN-89393-3, HN-30081-3, HN-89387-5	GQ149275, GQ199709, GQ245731	Histogenetics, Ossining, USA
B*150502	HN-92802-4	GQ245748	Histogenetics, Ossining, USA
B*1506	BY00560	GU138066	Carolyn K Hurley, Washington DC, USA
B*151002	HN-45679-7	GQ240387	Histogenetics, Ossining, USA
B*1535	JMDP01K067	AB536871	Kenji Tadokoro, Tokyo, Japan
B*1539	JMDP01K068	AB536872	Kenji Tadokoro, Tokyo, Japan
B*1561	BY00561	GU138065	Carolyn K Hurley, Washington DC, USA
B*1806	BY00559	GU138067	Carolyn K Hurley, Washington DC, USA
B*180702	HN-39607-9	GQ994065	Histogenetics, Ossining, USA
B*1833	HN-02964-7	GQ859551	Histogenetics, Ossining, USA
B*1835	HN-N96546, HN-N260491	GQ240386, GQ900552	Histogenetics, Ossining, USA
B*1847	HN-33661-9	GQ468246	Histogenetics, Ossining, USA
B*2720	BY00555	GU138071	Carolyn K Hurley, Washington DC, USA
B*2751	HN-62875-5	GQ900555	Histogenetics, Ossining, USA
B*2760	HN-90373-2	GQ149277	Histogenetics, Ossining, USA
B*350111	HN-21479-7, HN-39298-0	GQ345060, GQ401196	Histogenetics, Ossining, USA
B*350119	HN-83813-6	GQ900547	Histogenetics, Ossining, USA
B*350804	HN-25909-1	GQ449635	Histogenetics, Ossining, USA
B*3511	BY00556	GU138070	Carolyn K Hurley, Washington DC, USA
B*3521	JMDP36K043	AB536873	Kenji Tadokoro, Tokyo, Japan
B*3564	JMDP36K045	AB536874	Kenji Tadokoro, Tokyo, Japan
B*3598	HN-61859-0	GQ468249	Histogenetics, Ossining, USA
B*3717	HN-04271-5	GQ859545	Histogenetics, Ossining, USA
B*390110	HN-31356-8	GQ245750	Histogenetics, Ossining, USA
B*3912	BY00558	GU138068	Carolyn K Hurley, Washington DC, USA
B*3914	BY00557	GU138069	Carolyn K Hurley, Washington DC, USA
B*3915	BY00568	GU144507	Carolyn K Hurley, Washington DC, USA
B*400207	HN-N260568	GQ859532	Histogenetics, Ossining, USA
B*4011	JMDP01K063	AB537164	Kenji Tadokoro, Tokyo, Japan
B*4026	BY00569	GU256002	Carolyn K Hurley, Washington DC, USA
B*4029	JMDP36K042	AB537165	Kenji Tadokoro, Tokyo, Japan
B*4210	HN-21017-8	GQ468252	Histogenetics, Ossining, USA
B*440206	HN-N261934	GQ491085	Histogenetics, Ossining, USA
B*440209	HN-96331-0	GQ245739	Histogenetics, Ossining, USA
B*440210	HN-34788-2, HN-50429-6	GQ859550, GU017931	Histogenetics, Ossining, USA
B*4418	BY00554	GU138072	Carolyn K Hurley, Washington DC, USA
B*4474	HN-67794-6	GQ254352	Histogenetics, Ossining, USA
B*4486	HN-41994-1	GQ254335	Histogenetics, Ossining, USA
B*4496	HN-33582-4	GQ254349	Histogenetics, Ossining, USA
B*510110	HN-73186-2	GQ401191	Histogenetics, Ossining, USA
B*5165	HN-N94065, HN-25984-8, HN-N262510, HN-N035688, HN-N261989, HN-N262508	GQ245728, GQ449634, GQ859535, GQ914788, GQ914790, GQ914791	Histogenetics, Ossining, USA
B*5167	HN-75991-0	GQ245733	Histogenetics, Ossining, USA
B*5168	HN-62842-2	GQ914793	Histogenetics, Ossining, USA
B*5169	HN-77817-6	GQ240391	Histogenetics, Ossining, USA
B*5175	HN-N257741, HN-N263825, HN-N034218	GQ468247, GQ859531, GQ994058	Histogenetics, Ossining, USA
B*5182	HN-17946-0, HN-17947-8	GQ449643, GQ449644	Histogenetics, Ossining, USA
B*550105	HN-53197-2	GQ914792	Histogenetics, Ossining, USA
B*550106	HN-42409-1	GQ491084	Histogenetics, Ossining, USA
B*560102	HN-87472-3	GQ994062	Histogenetics, Ossining, USA
B*5602	BY00553	GU138073	Carolyn K Hurley, Washington DC, USA
B*570105	HN-59275-0	GQ859549	Histogenetics, Ossining, USA
B*5726	HN-94214-4	GQ859540	Histogenetics, Ossining, USA
B*5904	JMDP01K054	AB537166	Kenji Tadokoro, Tokyo, Japan
B*9553	HN-88641-3, HN-94111-9, HN-86738-9, HN-35520-8	GQ149278, GQ245745, GQ254341, GQ859547	Histogenetics, Ossining, USA
B*9581N	HN-95028-4	GQ245715	Histogenetics, Ossining, USA
Cw*010207	HN-81703-5, HN-27905-9, HN-28170-6	GQ180267, GQ180445, GQ254397	Histogenetics, Ossining, USA
Cw*010208	HN-02906-7, HN-55932-2, HN-53240-0, HN-35686-3, HN-57785-8, HN-99074-6, HN-87701-9	GU128019, GQ180241, GQ180248, GQ180389, GQ180411, GQ240487, GQ254390	Histogenetics, Ossining, USA
Cw*0110	BY00574	GU256007	Carolyn K Hurley, Washington DC, USA
Cw*0127	HN-35341-6	GU128046	Histogenetics, Ossining, USA
Cw*0132	HN-14507-0, HN-81436-2, HN-39457-9, HN-05381-9, HN-89122-6, HN-01820-8	GU017959, GQ161036, GQ180254, GQ180418, GQ180420, GQ240482	Histogenetics, Ossining, USA
Cw*020207	HN-16328-8, HN-11201-0, HN-83016-5, HN-56299-2, HN-75876-6	GU128039, GQ161064, GQ161067, GQ161069, GQ180442	Histogenetics, Ossining, USA
Cw*020208	HN-05717-7	GQ180244	Histogenetics, Ossining, USA
Cw*021602	HN-58658-8	GU017962	Histogenetics, Ossining, USA
Cw*0230	HN-19588-2, HN-09354-8	GQ161065, GQ994080	Histogenetics, Ossining, USA
Cw*0231	HN-42278-2	GQ180394	Histogenetics, Ossining, USA
Cw*030307	HN-18083-7	GU128023	Histogenetics, Ossining, USA
Cw*030308	HN-53315-0	GQ994082	Histogenetics, Ossining, USA
Cw*0321	BY00570	GU256003	Carolyn K Hurley, Washington DC, USA
Cw*033802	BY00571	GU256004	Carolyn K Hurley, Washington DC, USA
Cw*0374	HN-36900-8	GU128026	Histogenetics, Ossining, USA
Cw*0379	HN-22447-9	GQ994083	Histogenetics, Ossining, USA
Cw*040108	HN-69805-2, HN-03781-6	GU017945, GQ180269	Histogenetics, Ossining, USA
Cw*040108	AKB-651323	FN430727	Rainer Blasczyk, Hannover, Germany
Cw*040110	HN-15468-3, HN-11404-8, HN-80358-7, HN-63809-2, HN-03817-1	GU128038, GQ161049, GQ180259, GQ180270, GQ254388	Histogenetics, Ossining, USA
Cw*040113	HN-68703-0, HN-17952-4	GU017944, GU128022	Histogenetics, Ossining, USA
Cw*040115	HN-73887-7	GQ994086	Histogenetics, Ossining, USA
Cw*0439	HN-80328-7	GQ240486	Histogenetics, Ossining, USA
Cw*0448	HN-50961-3	GQ254395	Histogenetics, Ossining, USA
Cw*0452	HN-25199-0	GQ994087	Histogenetics, Ossining, USA
Cw*050107	HN-28304-6	GU017961	Histogenetics, Ossining, USA
Cw*050108	HN-65756-1	GU128049	Histogenetics, Ossining, USA
Cw*0526	N-671307	FN430728	Rainer Blasczyk, Hannover, Germany
Cw*0526	HN-08336-1	GQ240509	Histogenetics, Ossining, USA
Cw*0529	HN-54907-5	GQ180263	Histogenetics, Ossining, USA
Cw*0534	HN-27839-1	GU017940	Histogenetics, Ossining, USA
Cw*0623	HN-68335-1, HN-35876-3	GU128024, GQ161071	Histogenetics, Ossining, USA
Cw*0626	HN-74025-2, HN-72178-4	GQ180265, GQ254381	Histogenetics, Ossining, USA
Cw*0627	HN-28905-8	GQ180431	Histogenetics, Ossining, USA
Cw*070110	HN-62495-1	GQ161041	Histogenetics, Ossining, USA
Cw*070204	HN-66407-8	GQ180390	Histogenetics, Ossining, USA
Cw*070210	HN-45647-3, HN-04512-1, HN-25864-1, HN-20592-7	GU017943, GU128018, GQ161070, GQ994089	Histogenetics, Ossining, USA
Cw*070211	HN-13689-5	GQ180446	Histogenetics, Ossining, USA
Cw*0768	HN-53344-0, HN-28940-6, HN-76839-2, HN-91790-5, HN-40383-4	GU017950, GQ180252, GQ240505, GQ994073, GQ994084	Histogenetics, Ossining, USA
Cw*0773	HN-36623-6, HN-01145-2	GU128037, GQ240515	Histogenetics, Ossining, USA
Cw*0776	HN-38523-1	GQ161045	Histogenetics, Ossining, USA
Cw*0785	HN-24541-5	GQ161031	Histogenetics, Ossining, USA
Cw*0791	HN-39440-4, HN-55690-4, HN-27500-6	GQ161072, GQ180250, GQ240481	Histogenetics, Ossining, USA
Cw*0828	HN-13242-5, HN-30318-9, HN-48864-2	GU017957, GQ161061, GQ240491	Histogenetics, Ossining, USA
Cw*120308	HN-84987-5	GQ240483	Histogenetics, Ossining, USA
Cw*1227	HN-74768-3	GQ149306	Histogenetics, Ossining, USA
Cw*1414	HN-09762-5	GQ254392	Histogenetics, Ossining, USA
Cw*1507	BY00572	GU256005	Carolyn K Hurley, Washington DC, USA
Cw*1523	HN-30227-5	GQ240516	Histogenetics, Ossining, USA
Cw*160104	HN-N264212	GQ994077	Histogenetics, Ossining, USA
Cw*1614	HN-65879-2	GQ180444	Histogenetics, Ossining, USA
Cw*1615	HN-10995-9, HN-25373-9	GQ180434, GQ254384	Histogenetics, Ossining, USA
Cw*1616	HN-43823-8	GQ161050	Histogenetics, Ossining, USA
DRB1*133303	P-658554	FN430726	Rainer Blasczyk, Hannover, Germany
DPB1*1001	194229	FN594947	Klaus Witter, München, Germany
DPB1*110101	171880	FN598969	Klaus Witter, München, Germany
DPB1*3001	170324	FN594948	Klaus Witter, München, Germany

**Table 3:** Recently Published Sequences

Sequence	References
B*4088	(1)
B*5214	(2)
Cw*040110	(3)
Cw*0442	(4)
Cw*0624	(5)
Cw*0774	(6)
Cw*0766	(7)
Cw*0767	(7)
Cw*0824	(8)
DRB1*0337	(9)
DRB1*1533	(10)
DQB1*060105	(6)

All new and confirmatory sequences should now be submitted directly to the WHO Nomenclature Committee for Factors of the HLA System via the IMGT/HLA Database using the sequence submission tool provided. The IMGT/HLA Database may be accessed via the World Wide Web at: http://www.ebi.ac.uk/imgt/hla
